# Zingerone as a Neuroprotective Agent Against Cognitive Disorders: A Systematic Review of Preclinical Studies

**DOI:** 10.3390/ijms26136111

**Published:** 2025-06-25

**Authors:** Tosin A. Olasehinde, Oyinlola O. Olaokun

**Affiliations:** 1 Nutrition and Toxicology Division, Food Technology Department, Federal Institute of Industrial Research, Oshodi, P.M.B. 21023, Lagos 100261, Nigeria; 2 Department of Biochemistry, Genetics and Microbiology, School of Life Sciences, University of Kwazulu-Natal, Westville, Durban 4000, South Africa; 3 Department of Biology and Environmental Science, School of Science and Technology, Sefako Makgatho Health Science University, Pretoria 0208, South Africa; oyinlola.olaokun@smu.ac.za

**Keywords:** zingerone, zingiber officinalis, neuroinflammation, cognitive impairment, anxiety, depression, neurobehaviour, learning, memory, working memory

## Abstract

Cognitive problems are associated with impaired learning ability and memory dysfunction. Neuroinflammation has been identified as an important factor in the progression of anxiety and depressive disorders. Zingerone is a phenolic alkanone derived from ginger (Zingiber officinale Roscoe), which is known for its antioxidant and anti-inflammatory properties. A number of studies have investigated the effect of zingerone on neuroinflammation and cognitive impairment. However, this evidence has not been systematically reviewed. This study sought to systematically review the effect of zingerone on neuroinflammation and neurobehavioural changes associated with memory and learning impairment and anxiety-like and depressive-like behaviours. A systematic review was conducted using pre-defined search criteria on Google Scholar, Scopus and Web of Science. The records obtained were screened based on inclusion criteria, and data was extracted from the included studies. Out of the 482 studies that were identified, only 9 studies met the inclusion criteria. Neuroinflammatory markers such as interleukin 1β (IL-1β), interleukin 6 (IL-6), tumour necrosis factor-alpha (TNF-α) and ionized calcium binding adaptor molecule (IBA-1), as well as behavioural parameters including Morris water maze, Y-Maze, recognition test, passive avoidance test, elevated plus maze, sucrose preference test and forced swimming test were measured. Zingerone exhibited anti-neuroinflammatory effects by improving IL-1β, IL-6 and TNF-α levels. However, zingerone did not show any significant changes on activated microglia. The anti-neuroinflammatory mechanisms of zingerone were linked to the inhibition of nuclear factor kappa B (NF-kB) activation and the NOD-like receptor family, pyrin domain-containing 3 (NLRP3) inflammasome, as well as the reduction in neuronal nitric oxide synthase (nNOS). The anxiolytic and anti-depressive effects of zingerone were also associated with an improvement in cortical cholinergic transmission, the mitigation of oxidative stress and the upregulation of neurotransmitters such as serotonin and dopamine. This review provides scientific evidence on the cognitive enhancing and neuroprotective mechanisms of zingerone, which may be beneficial for future experimental investigations.

## 1. Introduction

Cognitive impairment caused by impaired learning and memory function, anxiety and depression has become a huge burden due to severe and long-lasting psychosocial difficulties. Previous reports suggest that 15–20% of elderly individuals above 65 have mild cognitive impairment [1]. Furthermore, 5–15% of individuals with mild cognitive impairment progressively develop dementia annually [2]. Impairment in cognitive function may be due to exposure to high levels of metals, microplastic or polycyclic aromatic hydrocarbons, metabolic disorders (diabetes), trauma in the brain or use of chemotherapeutic drugs [3,4,5]. Furthermore, ageing, including traumatic brain injury, neurodegeneration, stroke and infections in the central nervous system, may contribute to cognitive changes in elderly individuals. Moreover, cognitive impairments are also common in depressive and anxiety-like disorders. Some neuropsychological studies have shown the association between memory problems and major depression as well as anxiety disorders [6,7]. The work of Rock et al. [8] revealed that moderate impairment in executive function, memory and attention occurs in individuals experiencing depression. A better understanding of the pathological mechanisms involved in memory and learning impairment, depression and anxiety-like disorders will aid in providing therapeutic strategies for adequate treatment.

Many studies have shown that neuroinflammation may contribute to the etiology of memory problems, anxiety and depression [9,10,11]. An increase in inflammatory markers in the brain is associated with memory deficit, anxiety-like and depressive-like disorders [9]. Furthermore, the release of proinflammatory markers and activation of microglia has been identified in the brains of animals with anxiety and depressive disorder [9,12,13]. Moreover, microglia activation disrupts neuronal plasticity and memory function in the hippocampal region [14]. Hence, disruptions in the immune regulatory processes contribute to alterations and changes in cognitive function. Therefore, therapeutic strategies targeting activated microglia and inflammatory cytokines could mitigate impaired learning and memory function, depression and anxiety disorders.

*Zingiber offcinale* Roscoe (Ginger) is a common food spice that belongs to the Zingiberacae family. It is used in Ayurvedic medicine for the treatment of different ailments, including headache, pain, respiratory diseases, indigestion, fever, common cold and cough. The anti-inflammatory, gastroprotective, antihyperglycemic, antioxidant and anticancer activities of ginger have also been reported. Ginger contains volatile and non-volatile active compounds. Zingerone is one of the non-volatile compounds present in ginger. It contains a methoxy phenol group, which consists of a basic phenolic ring with a methoxy group that is attached to a benzene ring. It is commonly formed from gingerol [15,16]. It is primarily present in raw ginger; however, a significant amount may be formed during cooking and drying, which activates the conversion of gingerol to zingerone via a retroaldol reaction [16]. Several reports have shown the multiple pharmacological benefits of zingerone [17,18,19]. The antioxidant and anti-inflammatory properties of zingerone are linked to its radical scavenging capacity, which alters the expression of genes and improves inflammatory signals and pathways [16,19,20]. Some studies have also shown the effect of zingerone on neuroinflammatory markers, memory performance, and depressive-like behaviour [21,22]. In view of the search for alternative therapies to mitigate cognitive impairment associated with memory dysfunction, depression and anxiety-like disorders, a systematic review of the available results on the activity of zingerone will provide useful information for future studies. Hence, in this study, we summarized and discussed the therapeutic effects of zingerone on memory impairment and depressive- and anxiety-like disorders. We also discussed its effect on inflammatory cytokines and microglia as a possible mechanism of action against cognitive impairment associated with memory and learning impairment, anxiety and depressive disorders.

## 2. Materials and Methods

### 2.1. Search Strategy and Screening

A literature search for original articles in Scopus, Google Scholar and Web of Science was conducted using the following keywords: zingerone AND anxiety OR depression OR neuroinflammation OR cognitive OR memory OR neurodegeneration OR neurotoxicity. Articles that did not match the objectives of this study were screened out after a critical review of the title and abstract. Eligible studies were identified, and duplicates occurring from the different databases were eliminated. The eligible articles identified were further screened based on the inclusion criteria. In the screening process, articles that reported in vivo animal studies indicating the effect of zingerone on memory function, anxiety, depression and neuroinflammation were considered in this study. Animals induced with memory impairment, anxiety, depression or neuroinflammation and treated with zingerone were considered as the treatment group. Articles that did not use zingerone (pure compound) were excluded. In vitro and ex vivo studies were also excluded. Review papers were also not included.

### 2.2. Inclusion Criteria

Potential publications were thoroughly reviewed and included in this study if they met the following inclusion criteria:

1. Memory function was measured using associated behavioural parameters such as Morris water maze, Y-Maze and Recognition Memory.
2. Anxiety was measured using associated behavioural parameters such as an elevated plus maze, open field test and light/dark choice.
3. Depressive-like behaviour was measured using associated parameters such as forced swimming test, shock exposure (tail or foot shock), open space swimming test, sucrose consumption test, etc.
4. Neuroinflammation was measured using associated inflammatory markers, factors and pathways, including cytokines, chemokines, nitric oxide, NF-kB, NLRP3 inflammasome and Toll-like receptor-4 (TLR4).

### 2.3. Exclusion Criteria

During the screening, articles that did not involve the use of animal models (rat and mouse) were excluded. Studies that did not report measurement of markers and/or parameters associated with memory function, anxiety, depression and neuroinflammation were excluded. Also, studies in which the animals did not exhibit memory dysfunction, anxiety, depression and neuroinflammation were also excluded.

### 2.4. Data Extraction

Data were extracted from the included studies using the following study characteristics: (a) the author and year of publication, (b) details on zingerone intervention, including dose, route of administration and duration of the experiment, (c) information on the experimental model including the stressor/chemical agent used to induce pathology, dose, route of administration and duration of exposure, (d) the parameters evaluated or measured and (e) the main outcomes.

### 2.5. Quality Assessment of Included Studies

T.A.O. and O.O.O. assessed the quality of the methodology and experimental design of all the included studies. The quality assessment of the studies was performed using the Collaborative Approach for Meta-Analysis and Review of Animal Data from Experimental Studies (CAMARADES) checklist according to Zheng et al. [23], with slight modifications using ten different parameters, including (A) publication in a peer-reviewed journal; (B) statement of temperature control; (C) random allocation to groups; (D) allocation concealment; (E) blinded assessment of outcome; (F) avoidance of anesthetics with marked intrinsic neuroprotective properties; (G) appropriate animal disease models; (H) sample size calculation; (I) statement of compliance with regulatory requirements; and (J) statement regarding possible conflicts of interest. A point score was allotted to a study if it met the requirement of each parameter stated. A zero-point score was given if the details of the study design did not meet the requirement of the stated parameter. From the scores given, study designs from each included study were rated on a scale of 0 (lowest) to 10 (highest).

## 3. Results

### 3.1. Article Selection and Study Characteristics

**Figure 1:**
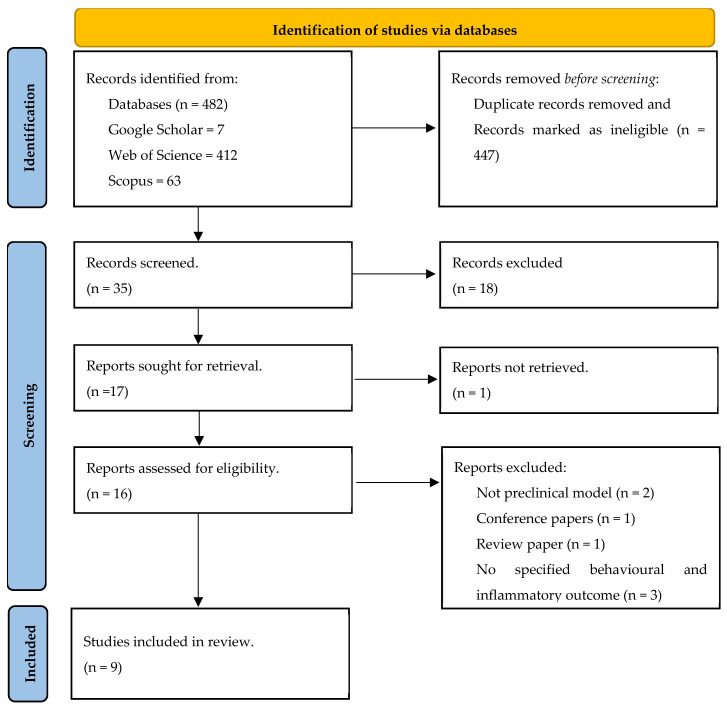
PRISMA flow diagram of article selection.

As shown in Figure 1, 482 records were obtained from Web of Science, Scopus and Google Scholar. After removing ineligible documents and duplicates, 35 articles were further screened for eligibility, and records that did not report in vivo animal studies were removed. Furthermore, 17 articles were screened based on the inclusion criteria, and 7 articles were excluded because the following applied: (i) it was a review paper, (ii) it was a conference paper, (iii) it was not specific about the behavioural and inflammatory outcomes or (iv) it did not use a preclinical model. Nine articles were included in this systematic review.

Table 1 reveals the nine included studies as specified by the inclusion criteria. These studies were published between 2015 and 2023. Out of the nine studies, six used Wistar rats, two studies used mice, and one study used Sprague Dawley rats. All the studies used male animals except one study that did not provide information regarding the sex of the animals. Apart from two studies that used a stress model to induce anxiety and depression in the rats, other studies used different agents such as 1-methyl-4-phenyl-1,2,3,6-tetrahydropyridine (20 mg/kg), cadmium (5 mg/kg), glioma cells, lithium and pilocarpine, ketamine and sodium arsenite. These agents were administered at different doses. While one study used the oral route to administer cadmium, four other studies used the intraperitoneal route. Only one study used intracerebroventricular injection of C6 glioma cells, while other studies employed a stress-induced model.

### 3.2. Quality Assessment of Methods and Experimental Models of Included Studies

Table 2 revealed the results of the quality of the included studies using the CAMARADES checklist. Our findings revealed that all the studies were published in peer-reviewed journals, used appropriate animal disease models and indicated a statement of compliance with regulatory requirements. However, none of the studies showed proof of sample size calculation and blinded assessment of outcome. Also, only one study revealed the allocation of concealment. Eight studies also showed avoidance of anesthetics with marked intrinsic neuroprotective properties and statements regarding possible conflicts of interest.

### 3.3. Effect of Zingerone on Neuroinflammatory Markers

The included studies revealed the effect of zingerone on some proinflammatory cytokines. Two studies revealed that zingerone significantly reduced IL-6 levels, which were elevated in the brain of chronic unpredictable mild-stress rats and LiCl-and-Pilocarpine-induced epileptic mice. Three studies also reported an increase in IL-1β in the brains of the animals. However, treatment with zingerone significantly reduced IL-1β levels in the brains of epileptic animals compared to the diseased control. Furthermore, four studies reported elevated levels of TNF-α in the brains of the animals. Treatment with Zingerone also significantly reduced TNF-α levels in the brains of the animals.

Two studies also reported an upregulation of NF-kB expression in the brain tissues of the animals. However, treatment with zingerone triggered a downregulation of NF-kB expression. Only one study reported the effect of zingerone on NO and nNOs in the brains of the studied animals. NO levels and nNOs expression were significantly elevated in the animals. However, treatment with zingerone reduced NO levels and nNOs expression. The effect of zingerone on the NLRP3 inflammasome was also reported in one included study. Zingerone inhibited the NLRP3 inflammasome, which was significantly high in morphine-treated mice brains. One of the included studies also showed the effect of zingerone on IBA-1 and GFAP expression in a 1-methyl-4-phenyl-1,2,3,6-tetrahydropyridine (MPTP) mouse model. MPTP activated microglia cells and astrocytes in the striatum and substantia nigra. It was observed that zingerone did not exhibit any significant changes in the microglia cells and astrocytes, as revealed by IBA-1 and GFAP expression.

### 3.4. Zingerone and Memory Function

Two studies reported the effect of zingerone on spatial working memory and exploratory behaviour using the Y-Maze test. Treatment with zingerone significantly increased spatial activity, which was revealed by an increase in time spent in the novel arm, the number of arm entries and alternation in memory-impaired animals. Two studies also reported the effect of zingerone on spatial memory and learning using the Morris water maze test. While one of the studies revealed that pretreatment with zingerone reduced escape latency and increased time spent in the target quadrant in lithium chloride and pilocarpine-induced cognitively impaired rats, the other study showed that zingerone did not show any effect on cognitive function in chronic restraint stress rats. Furthermore, only one study reported the effect of zingerone on spatial learning using a novel recognition test. Zingerone (50 and 100 mg/kg) improved the recognition index of memory-impaired rats. In the passive avoidance test, which was reported by only one included study, the result showed that zingerone improved cognitive function in memory-impaired rats through an increase in retention time and a decrease in acquisition time.

### 3.5. Effect of Zingerone on Anxiety and Depressive-like Disorders

One study reported the effect of Zingerone on anxiety-like behaviour using an elevated plus maze. Anxiety levels were assessed based on time spent and the number of entries into the open arms of the maze. In animals with anxiety-like behaviour due to infection with glioma cells, an increase in time spent in the open arms, as well as an increase in the number of open-arm entries, was observed. However, treatment with zingerone significantly reduced the time spent in the open arms and the number of open-arm entries.

The effect of zingerone on depressive-like behaviour was reported in two included studies using sucrose preference tests and forced swimming tests. In animals with depressive-like behaviour, a significant decrease in sucrose consumption and an increase in immobility time in forced swimming tests was observed. However, zingerone increased the percentage of sucrose consumption and reduced immobility time in memory-impaired animals.

## 4. Discussion

In this study, we systematically examined the therapeutic effects of zingerone against neuroinflammation and neurobehavioural impairment associated with memory dysfunction, anxiety and depressive-like behaviour. To the best of our knowledge, this is the first systematic review on the effect of zingerone on neuroinflammation and neurobehavioural function in preclinical studies. Zingerone is one of the active components of ginger, a potent medicinal food with several biological activities. This study included studies that reported the effect of zingerone on neuroinflammation and behavioural parameters associated with memory and learning, anxiety and depressive-like behaviour. Studies involving extracts containing zingerone or mixtures were not included. Hence, the few reports identified in this study suggest that more research is required to investigate the therapeutic effect of zingerone and its exploration as a novel drug to mitigate neuroinflammation and neurobehavioural deficit, especially in neurodegenerative diseases.

Neuroinflammation is an important characteristic of most neurodegenerative diseases and has been identified as a critical contributory factor to cognitive dysfunction [31]. Studies have shown that changes in inflammatory and immune responses may induce impairment in memory function, anxiety and depressive-like behaviour [32,33]. The administration of zingerone showed promising results by reducing levels of proinflammatory markers in the brains of studied animals. High levels of TNF-α, IL-6 and IL-1β were observed in memory-impaired rats [29]. However, treatment with zingerone reduced levels of TNF-α, IL-6 and IL-1β in rats’ brains. Zingerone also reduced TNF-α and IL-6 levels in chronic unpredictable mild stress-induced depressive-like behaviour in rats [27]. Yilmaz, Gur, Kucukler, Akaras and Kandemir [30] reported that zingerone reduced TNF-α and IL-1β levels in sodium arsenite-induced neuroinflammation in rats. The persistent release of proinflammatory cytokines such as TNF-α, IL-6 and IL-1β is an indication of inflammatory and pathological changes. High levels of inflammatory cytokines may impair the blood–brain barrier and induce rupture, trigger the upregulation of adhesion molecules and induce the diffusion of toxic molecules into the brain [34,35]. These cytokines, especially TNF-α, can also induce neuronal apoptosis via the activation of receptors and caspase-3-mediated pathways [36,37].

One of the molecular mechanisms of the anti-neuroinflammatory effect of zingerone observed in the included studies is the inhibition of NF-kB activation in the brain. The NF-kB extends the inflammatory response via the activation of the production of inflammatory cytokines [38]. NF-kB modulates neuronal activities in the brain, thereby regulating the transcription of genes for proinflammatory cytokines and chemokines [39]. The activation of NF-kB-induced neuroinflammation was observed in the brains of rats induced with sodium arsenite [30]. However, zingerone mitigated neuroinflammation induced by sodium arsenite via the downregulation of NF-kB expression. The co-administration of lithium and pilocarpine triggered the upregulation of NF-kB expression in a mouse brain, suggesting the activation of inflammatory pathways, the production of proinflammatory markers and neuroinflammation [29]. Treatment with zingerone significantly reduced NF-kB expression, thereby mitigating inflammatory changes in the brain.

From two included studies, the effect of zingerone on two important inflammatory factors—NO and nNOS—were also observed. Previous studies have implicated nitrosative stress as a contributory factor to neuroinflammation [40,41]. Hence, an anti-nitrosative therapy can combat neuroinflammation. NO is produced from neuronal nitric oxide synthase (nNOS) and aids in regulating inflammatory and immune responses [42]. The work of Maleki, Moosavi, Zeidooni, Azadnasab and Khodayar [28] showed that the administration of ketamine increased NO levels, which correlated with the increase in TNF-α expression. The administration of sodium arsenite could trigger neuroinflammation via the upregulation of nNOS expression, which correlated with an increase in TNF-α, IL-1β and NF-kB expressions in rats’ brains [30]. However, treatment with zingerone reduced NO levels and nNOS expression, thus preventing neuroinflammation triggered by ketamine and sodium arsenite.

Another notable anti-neuroinflammatory mechanism of zingerone is its inhibitory effect on the NLRP3 inflammasome. The disruption of the regulation of NLRP3 inflammasome formation and activation has been implicated in neuroinflammation and the pathogenesis of some neurodegenerative diseases [43]. The NLRP3 inflammasome is a protein capable of sensing danger signals; hence, it is formed and activated in response to immune inflammatory response signals [44]. Therefore, when the inflammasome is formed, it triggers the activation of proinflammatory protease caspase-1 and pyroptosis, which in turn initiates the cleavage and activation of proinflammatory cytokines. The work of Molavinia, Nikravesh, Pashmforoosh, Vardanjani and Khodayar [22] revealed that zingerone suppressed NLRP3 inflammasome activation and inhibited the cleavage of caspase and IL-1β from the multiprotein complex. This action may contribute to the suppression of cytokine production and the mitigation of neuroinflammation.

Microglia-target therapeutic interventions and treatment have become a novel strategy for combating neuroinflammatory disorders or neurodegenerative conditions associated with neuroinflammation. Microglia cells are macrophage-like cells and immune sentinels that regulate inflammatory responses in the brain [39]. Persistent activation of microglia may induce chronic neuroinflammatory response and an overproduction of inflammatory markers, which may, in turn, trigger neuroinflammation and neurodegeneration [45]. In the work of Choi, Bae, Park and Jeong [24], the effect of zingerone on microglia cells in rat striatum and substantia nigra was reported. The results showed activated microglia cells and astrocytes in the striatum and substantia nigra in a 1-methyl-4-phenyl-1,2,3,6-tetrahydropyridine mouse model. Interestingly, zingerone did not show any significant changes in the number of microglia cells, which suggests that it had no effect on microglia activation and neuroinflammation induced by 1-methyl-4-phenyl-1,2,3,6-tetrahydropyridine. The reason for the observed results is not clear, however many factors regarding the study design may also contribute to the results obtained. Hence, there is a need for more studies to be conducted on the effect of zingerone on activated microglia cells and astrocytes. The results from the study of Choi, Bae, Park and Jeong [24] will be beneficial for future studies. The dosage of zingerone (10 mg/kgbw) used in their study may likely contribute to the absence of changes observed in microglia cells. Also, the duration of the study was short, involving the administration of zingerone for 3 days, with a total study duration of 7 days.

Two experimental investigations revealed the effect of zingerone on learning and memory deficit induced by pilocarpine administration and chronic stress using the Morris water maze test. In a study conducted by Rashid et al. [29], zingerone improved learning and memory in cognitively impaired rats administered with lithium and pilocarpine. The improvement in cognitive function exerted by zingerone could be associated with improved neurochemical signalling and neuroprotection against neuronal damage. In the same study, cognitively impaired rats treated with zingerone showed improved cholinergic transmission and antioxidant status, inhibited the expression of inflammatory mediators and mitigated neuroinflammation, which may account for the improvement in learning and memory function. However, the work of Upadhyaya, Sharma, Akhtar and Pilkhwal Sah [21] showed that zingerone did not improve memory function in chronically stressed rats, although it exerted positive significant changes in other neurobehavioural parameters. This could be due to the dosage of zingerone used in the study. Other factors, including the duration of the experiment and the route of administration, may also affect these results. Hence, there is a need for further studies to explore higher doses of zingerone and different experimental models.

The effect of zingerone treatment on anxiety-like behaviour was investigated in glioma cell-induced anxiety in a rat model. Infection with glioma cells triggered anxiety-like behaviour, as revealed by the elevated plus maze task through a reduction in time spent and a decrease in open arm entries in the open arm of the maze. However, treatment with zingerone significantly triggered anxiolytic effects by increasing time spent in the open arm as well as increasing open arm entries in the elevated plus maze. The anxiolytic effect of zingerone is associated with the regulation of the cortical cholinergic system and the mitigation of oxidative stress. Moreover, the molecular mechanisms of the anxiolytic effects of zingerone need to be further explored. More studies should aim to unravel the effect of zingerone on anxiety.

The sucrose preference and forced swimming tests were used to evaluate the effect of zingerone on depressive-like behaviour. Treatment with zingerone significantly increased sucrose consumption in the stressed rats. Zingerone also reduced immobility time in depressed animals. The results from the two studies revealed that zingerone exhibited anti-depressant activities. Also, the reduction in immobility time in forced swimming tests is associated with anti-depressant effects. One of the essential factors that contribute to a reduction in immobility time in depressive animals is associated with the levels of neurotransmitters in the brain. Hence, impairment in neurotransmission systems may contribute to the development of depression. The work of Li et al. [46] revealed that levels of serotonin and dopamine were significantly reduced in different brain regions (cortex, amygdala, hippocampus and hypothalamus) in animals with depressive behaviour. The level of 5-HT was significantly reduced in stress-induced depressive animals [27]. Also, levels of dopamine, norepinephrine and serotonin were also altered in the brain of depressed rats [47]. The serotonergic, adrenergic and dopaminergic systems are important targets for the treatment of depression. Some serotonin receptors, such as the 5-HT, dopaminergic and adrenergic receptors control depressive progression and are target markers for drugs used for the treatment of depression. Hence, the anti-depressive effect of zingerone could be linked to the modulation of some neurotransmitters and their receptors in depressive-like animals. The report of Jadhav and Sable [27] showed that zingerone at a high dose of 250 mg/kg improved the levels of 5-HT in rats with depressive behaviour. Furthermore, zingerone also increased levels of dopamine, which was depleted by hydroxydopamine in the striatum of depressed animals. There are very few studies on the effect of neurotransmitters that have been linked to depressive-like behaviour. The effect of zingerone on 5-HT, dopamine and norepinephrine in different brain regions should be explored.

Though this study has revealed the importance and benefit of zingerone for the mitigation of neuroinflammation and depression in preclinical studies, some limitations need to be considered to explain the results obtained from this systematic review. One of the key limitations of this study is the variation in the animal model and design of the experimental investigation in the individual studies, which could make pooling the outcome of this study cumbersome. These variations in the included studies affected the heterogeneity of the results. Another limitation of this study is that no specific brain region was selected as part of the selection criteria. The included studies showed that the markers selected were in different brain regions, or the whole brain was used in the investigation. Furthermore, sex differences were also not considered. There were variations in the dose of zingerone, and the period or length of the experiment also varied in the selected studies. Also, this systematic review depended on the outcomes and results of published studies on the effect of zingerone on neuroinflammation and behavioural function. The limited studies used in the study are also noteworthy, which points to the fact that more studies are required to establish the therapeutic effect and molecular mechanisms of zingerone.

## 5. Conclusions

The goal of this systematic review is to assess the impact of zingerone on neuroinflammation, and behavioural impairment associated with learning and memory and anxiety-like and depressive-like behaviour. Our findings from the included studies showed that zingerone holds good therapeutic potential and could be beneficial against neuroinflammation and memory and learning impairment, anxiety and depression based on preclinical studies. One of the key contributory factors to the therapeutic action of zingerone is its interaction with neuroinflammatory markers such as IL1-β, IL-6 and TNF-α. Zingerone was able to mitigate neuroinflammation by inhibiting NF-kB activation and NLRP3 inflammasome, modulating NO and nNOs expression. Furthermore, zingerone improved learning and memory and triggered anxiolytic and anti-depressive effects via improved cholinergic transmission, antioxidant status and modulation of the levels of serotonin and dopamine. However, future research should prioritize the effect of zingerone on neurotransmitters associated with zingerone in different and specific brain regions. A dose–response study is required to ascertain the efficacy of zingerone. Also, the effect of zingerone on microglia should be explored. Ultimately, future clinical trials need to be considered to translate the results to the human context.

## Figures and Tables

**Table 1:** Study characteristics and main outcomes from included studies.

Study	Zingerone Intervention (Dose, Route of Administration and Duration of Experiment)	Experimental Model	Evaluated Parameters	Main Outcomes
Choi et al. [24]	10 mg/kg/day intraperitoneal, 10 days	1-methyl-4-phenyl-1,2,3,6-tetrahydropyridine mouse model (20 mg/kg, i.p. injection). Male C57BL/6 mice (10 weeks old).	IBA-1 and GFAP expression	No significant changes in IBA-1 and GFAP expression
Anyanwu et al. [25]	200 mg/kg/day oral administration, 21 days	Cadmium-induced model (5 mg/kg, oral administration). Male Wistar rats.	Behaviour—Y-Maze	A significant increase in spatial activity, evident by an increase in time spent in the novel arm, number of arm entries, alternation and percentage alternation
Chopra et al. [26]	100 mg/kg/day oral administration, 42 days	Brain-inflicted glioma cells in Male Wistar rats. Intracerebroventricular injection of C6 glioma cells (1 × 10^5^/10 µL.	Y-Maze Passive avoidance test Novel object Recognition test Elevated plus maze	Improved exploration behaviour, improved learning and memory ability, anti-anxiety-like behaviour
Jadhav et al. [27]	250 mg/kg, 21 days oral administration	Chronic upredictable mild stress rats. Wistar rats.	Sucrose preference test Forced swimming test IL-6, TNF-α	Improved anti-depressant activity Reduced IL-6 and TNF-α
Maleki et al. [28]	50 mg/kg, 14 days, oral administration	Ketamine-induced manic-like behaviour. Ketamine (25 mg/kg, i.p.). Male Wistar Rats.	NO TNF-α	Reduced NO levels Reduced TNF-α levels
Molavinia et al. [22]	50, 100 and 200 mg, oral gavage, 7 days	Morphine (10 mg/kg, i.p.)-induced oxidative stress and inflammasome in mice brain. Adult male NMRI mice.	IL-1β NLRP3	Reduced IL-1β levels Inhibited NLRP3 inflammasome
Rashid et al. [29]	25 and 50 mg/kg, oral administration for 15 days	Lithium (3 m/Eq/kg, i.p.) and Pilocarpine (10 mg/kg i.p.)-induced status epilepticus in mice. Male Swiss Albino mice.	Morris water maze NF-kB IL-1β IL-6 TNF-α	Reduced escape latency, increase time spent in target quadrant and improved memory and learning. Reduced NF-kB, IL-1β, IL-6 and TNF-α levels
Upadhyaya et al. [21]	25, 50 and 100 mg/kg oral gavage for 28 days	Stress-induced depressive-like behaviour in male Wistar rats.	Morris water maze Forced swimming test Sucrose preference test	Increased sucrose consumption Increased immobility time Zingerone did not exhibit any effect on memory function
Yilmaz et al. [30]	25 and 50 mg/kg oral administration for 14 days	Sodium arsenite-induced sciatic nerve damage and neuroinflammation in male Sprague Dawley rat brain. Sodium arsenite (10 mg/kg).	NF-kB IL-1β TNF-α nNOS	Reduced expression and downregulation of NF-kB, IL-1β TNF-α and nNOS

**Table 2:** Quality assessment of study designs of included studies.

Study	A	B	C	D	E	F	G	H	I	J	Total
Choi et al. [24]	1	0	1	0	0	0	1	0	1	1	5
Anyanwu et [25]	1	0	1	0	0	1	1	0	1	0	5
Chopra et al. [26]	1	0	1	0	0	1	1	0	1	1	6
Jadhav et al. [27]	1	1	1	1	0	1	1	0	1	1	8
Molavinia et al. [22]	1	1	1	0	0	1	1	0	1	1	7
Maleki et al. [28]	1	1	1	0	0	1	1	0	1	1	7
Rashid et al. [29]	1	1	1	0	0	1	1	0	1	1	7
Upadhyaya et al. [21]	1	1	1	0	0	1	1	0	1	1	7
Yilmaz et al. [30]	1	1	0	0	0	1	1	0	1	1	6

(A) Publication in peer-reviewed journal; (B) statement of temperature control; (C) random allocation to groups; (D) allocation concealment; (E) blinded assessment of outcome; (F) avoidance of anesthetics with marked intrinsic neuroprotective properties; (G) appropriate animal disease models; (H) sample size calculation; (I) statement of compliance of regulatory requirements; (J) statement regarding possible conflicts of interest.

## Data Availability

Not applicable.
